# Treatment of Failed Mitral Valve Bioprostheses or Repairs: Transcatheter Mitral Valve-in-Valve/Valve-in-Ring Versus Redo Surgical Mitral Valve Replacement

**DOI:** 10.3390/jcm15218423

**Published:** 2026-10-29

**Authors:** Jacqueline Kruse, Eissa Alaj, Nihal Wilde, Hossien Alirezaei, Marwan Hamiko, Sebastian Zimmer, Marcel Weber, Enzo Lüsebrink, Miriam Silaschi, Farhad Bakhtiary

**Affiliations:** 1 Department of Cardiac Surgery, University Hospital Bonn, 53127 Bonn, Germany; eissa.alaj@ukbonn.de (E.A.); hossien.alirezaei@ukbonn.de (H.A.); marwan.hamiko@ukbonn.de (M.H.); miriam.silaschi@ukbonn.de (M.S.); farhad.bakhtiary@ukbonn.de (F.B.); 2 Department of Cardiology, Bundeswehr Central Hospital Koblenz, 56072 Koblenz, Germany; nihal.wilde@gmx.de; 3 Department of Cardiology, University Hospital Bonn, 53127 Bonn, Germany; sebastian.zimmer@ukbonn.de (S.Z.); marcel.weber@ukbonn.de (M.W.); enzo.luesebrink@ukbonn.de (E.L.)

**Keywords:** valve-in-valve, valve-in-ring, bioprostheses, mitral valve degeneration, redo mitral valve replacement, transcatheter mitral valve replacement

## Abstract

**Background/Objectives:** Transcatheter mitral valve-in-valve (ViV) and valve-in-ring (ViR) procedures have emerged as alternative treatment options for failed mitral valves following prior bioprosthetic replacement or ring annuloplasty. However, redo surgical mitral valve replacement (redo-MVR) remains the standard therapy despite its perceived higher procedural risk. This study aimed to descriptively compare short- and long-term outcomes of ViV/ViR and redo-MVR. **Methods:** We retrospectively included patients older than 60 years who underwent treatment of failed mitral valve bioprostheses or repairs between 2000 and 2022 (*n* = 30; ViV/ViR cohort: 2008–2022). Patients treated with ViV/ViR (*n* = 15) were compared with those undergoing redo-MVR (*n* = 15). Endpoints were reported according to Mitral Valve Academic Research Consortium (MVARC) criteria. Technical, device, and procedural success were defined according to the MVARC criteria and applied identically to both groups. Owing to the small sample size and substantial baseline differences, this study is descriptive and hypothesis-generating. **Results:** Patients in the ViV/ViR group were significantly older than redo-MVR patients (76.9 ± 5.8 vs. 68.0 ± 8.4 years, *p* < 0.001). EuroSCORE II did not differ significantly between groups. Thirty-day mortality was comparable, with one death in the ViV/ViR group and none after redo-MVR (6.7% vs. 0%, *p* = 1.00). Device success at 30 days was significantly higher after redo-MVR compared with ViV/ViR (86.7% vs. 40.0%, *p* = 0.02). Neurological events were rare and similar between groups. Degenerated bioprostheses were more frequent in the ViV/ViR group (86.6%), whereas prior annuloplasty ring implantation predominated in redo-MVR patients (80.0%, *p* < 0.01). Pre-procedural and discharge mitral valve gradients were higher in ViV/ViR patients. Implanted valve size was smaller in the ViV/ViR cohort (28.2 ± 1.4 vs. 29.5 ± 1.8 mm, *p* = 0.03). **Conclusions:** In this small, retrospective, single-center cohort with substantial baseline differences (e.g., age), 30-day mortality was low in both groups. Device success was higher after redo-MVR, mainly because of higher postoperative gradients after ViV/ViR. These findings are descriptive and hypothesis-generating and do not constitute evidence of comparative treatment effectiveness. Both approaches may be considered complementary options guided by a multidisciplinary heart team.

## 1. Introduction

The management of aortic valve disease has undergone a revolutionary change as a result of the development of transcatheter procedures for the treatment of valvular heart disease, which have become the gold standard therapy in patients with increased surgical risk and are being considered across a broader spectrum of risk profiles [1,2,3]. With favorable clinical outcomes, this approach was also employed to treat patients with failing bioprostheses in the aortic position, so-called valve-in-valve (ViV) therapy [4,5]. It was then applied to additional anatomical regions, such as the mitral valve (MV) [6,7].

The use of ViV or valve-in-ring (ViR) procedures for transcatheter treatment of patients with failed mitral annuloplasty rings or bioprostheses will be of increasing interest in the future due to the growing use of bioprostheses in younger patients and the increasing number of successful repairs [8,9,10]. Additionally, the Mitral Valve Academic Research Consortium (MVARC) has established standardized endpoint definitions and recommendations in order to guide the evaluation of transcatheter treatments for MV disease and improve consistency in the comparative effectiveness of published data [11].

Outcome data of ViV/ViR procedures should be compared to the gold standard of repeat mitral valve replacement (redo-MVR) [1] to inform heart teams and determine their future potential, despite the fact that these new techniques are still reserved for high-risk patients in the context of MV treatment. For this reason, we evaluated short- and long-term patient outcomes for both therapy modalities using the MVARC criteria [11], in a descriptive, hypothesis-generating analysis.

## 2. Materials and Methods

### 2.1. Patients

This was a retrospective observational study. From 2008 to 2022, all patients who underwent mitral valve-in-valve (ViV) or valve-in-ring (ViR) procedures for failed mitral valve (MV) bioprostheses or previous MV repairs and who fulfilled the predefined eligibility criteria were retrospectively identified from the institutional database and included in the ViV/ViR cohort (*n* = 15).

For the surgical comparison cohort, the institutional database was screened for all patients who underwent redo mitral valve replacement (redo-MVR, i.e., surgical explantation of a failed bioprosthesis or annuloplasty ring and implantation of a new prosthesis; “MVR” always denotes mitral valve replacement; previous mitral valve repairs are referred to as annuloplasty rings) between 2000 and 2022. During this period, a total of 116 redo-MVR procedures were performed.

Patients aged 60 years or younger (*n* = 64) at the time of reoperation and patients with infective endocarditis (*n* = 37) were excluded. The remaining eligible patients resulted in a final redo-MVR comparison cohort of 15 patients.

The 15 patients in the redo-MVR comparison cohort therefore represent a predefined subset of the complete cohort of 116 redo-MVR procedures performed during the study period and were not selected based on clinical outcome.

Treatment strategy was determined by a multidisciplinary heart team. The valve-in-valve application for mitral valves [12], together with measurements obtained by three-dimensional echocardiography and computed tomography, was used to assess valve dimensions and determine appropriate valve sizing.

Given the limited number of patients fulfilling the predefined eligibility criteria, individual one-to-one matching between the ViV/ViR and redo-MVR cohorts was not feasible. Both cohorts were therefore defined according to the predefined eligibility criteria and comprised patients who actually underwent the respective procedures during the specified study periods.

The study was reviewed and approved by the Ethics Committee of the University Hospital of Bonn (Ethics Application No. 134/22). The patients were followed up longitudinally.

**Figure 1:**
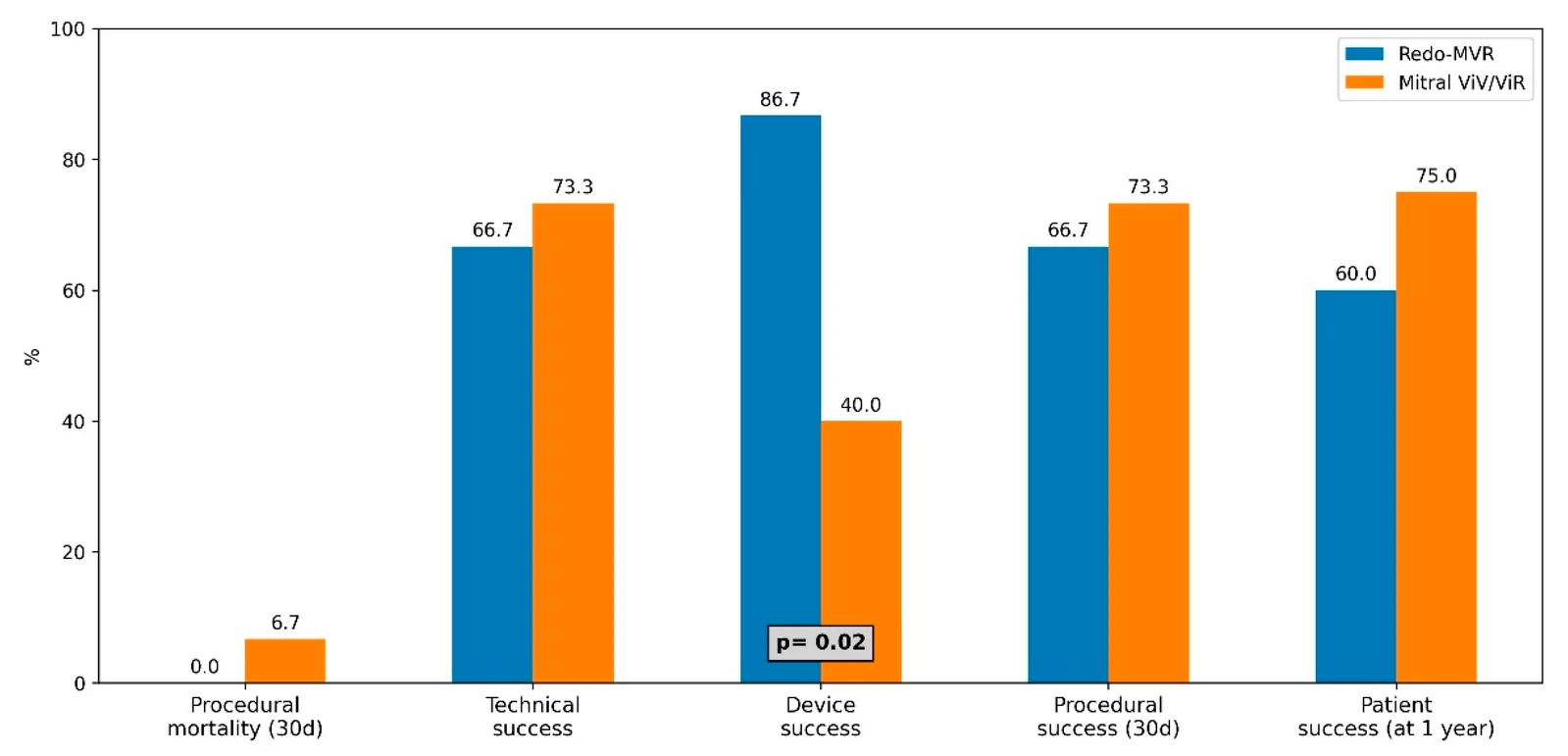
Study design and results according to the MVARC endpoints. ViV/ViR = valve-in-valve/valve-in-ring; MVR = mitral valve replacement; MVARC = Mitral Valve Academic Research Consortium.

The study design is summarized in Figure 1.

### 2.2. Endpoints

Outcomes were compared according to MVARC criteria [11], which were applied identically to both cohorts, i.e., to the implanted prosthesis irrespective of whether it was delivered by a transcatheter (ViV/ViR) or a surgical (redo-MVR) approach. The relevant MVARC endpoints were technical success, device success, and procedural success, which are defined hierarchically as follows: Technical success (at exit from the procedure room) was defined as the patient being alive, successful access and delivery of the prosthesis (including retrieval of the delivery system in ViV/ViR), successful deployment and correct positioning of the first intended prosthesis, and freedom from emergency surgery or reintervention related to the device or access procedure. Device success (assessed at 30 days) was defined as absence of procedural mortality or stroke, proper placement and positioning of a single prosthesis in the intended anatomical location, freedom from unplanned surgery or intervention related to the device or access procedure, and intended performance of the prosthesis, i.e., no prosthesis migration or embolization, no left ventricular outflow tract obstruction, no moderate or severe (grade II/III) mitral regurgitation (transvalvular or paravalvular), and a mean transmitral gradient < 5 mmHg. Procedural success was defined as device success together with the absence of major procedure- and prosthesis-related adverse events (all-cause mortality, stroke, life-threatening bleeding, major vascular complications, major cardiac structural complications, and acute kidney injury stage 2/3 within 30 days, as defined by MVARC). Thus, procedural success could only be achieved among patients who fulfilled the criteria for device success. The two endpoints are therefore nested and not circular: device success is defined by procedural mortality/stroke and by the position and performance of the prosthesis, whereas procedural success additionally requires freedom from the other major clinical adverse events. For redo-MVR, the “device” is the surgically implanted bioprosthesis, and “access” refers to the surgical access (sternotomy or thoracotomy); device failure at 30 days thus denotes early failure of the new prosthesis or its surgical access to meet the criteria above and not late structural valve degeneration. MVARC bleeding, neurological, renal, and other clinical endpoint definitions were applied where appropriate [11].

### 2.3. Statistical Analysis

For categorical variables, data are given as absolute numbers and percentages, and for continuous variables, as mean values and standard deviations. Continuous variables were compared using paired and unpaired *t*-tests, whereas dichotomous variables were compared using Fisher’s exact test. Missing values were not replaced. The threshold for significance was two-tailed *p* < 0.05. Kaplan–Meier analyses were used to calculate survival, and the log-rank test was used to compare results. All calculations were carried out using GraphPad Prism 5.0 (GraphPad Software Inc., La Jolla, CA, USA).

## 3. Results

The previously indicated inclusion criteria were met by 15 ViV/ViR patients and 15 redo-MVR patients, who were subsequently analyzed. Patients in the ViV/ViR group were significantly older than those in the redo-MVR group (76.9 ± 5.8 vs. 68.0 ± 8.4 years, *p* < 0.001). Sex distribution was comparable between groups (*p* = 0.46). Logistic EuroSCORE II (ViV/ViR: 12.0 ± 2.9 vs. redo-MVR: 10.0 ± 2.7, *p* = 0.06) did not differ significantly between groups, although a trend toward higher EuroSCORE II values was observed in the ViV/ViR cohort. All patients in both groups were in NYHA class III or IV. The prevalence of peripheral vascular disease was similar between groups (*p* = 1.00). Compared with redo-MVR patients, glomerular filtration rate was significantly lower in the ViV/ViR group (*p* < 0.01). Although the ViV/ViR group included numerically more patients with a history of stroke or transient ischemic attack (TIA), this difference was not statistically significant (*p* = 0.35).

Degenerated biological prostheses were more frequent in the ViV/ViR group (86.6%), whereas patients in the redo-MVR group more commonly had a previous annuloplasty ring implantation (80.0%, *p* < 0.01). Time since previous mitral valve surgery was significantly longer in the ViV/ViR group (*p* < 0.01).

Mitral valve dysfunction was classified as stenosis, regurgitation, or mixed type. In the ViV/ViR group, mixed valve dysfunction was most common, whereas in the redo-MVR group mitral regurgitation predominated. However, this difference was not statistically significant (*p* = 0.18). Baseline mean transvalvular gradients were significantly higher in ViV/ViR patients (*p* = 0.05). Baseline characteristics are summarized in Table 1.

Procedural approaches differed markedly between groups, with ViV/ViR performed via transapical access in 6 patients (40%) or transfemoral access in 9 patients (60%), while redo-MVR was predominantly conducted via sternotomy (93.3%). In the ViV/ViR group, Sapien 3 valves were most commonly used (80%). Implanted valve size was significantly smaller in the ViV/ViR group compared to redo-MVR (28.2 ± 1.4 mm vs. 29.5 ± 1.8 mm; *p* = 0.029). Cardiopulmonary bypass and cross-clamp times were only applicable to redo-MVR. The need for postoperative mechanical circulatory support was low and comparable between groups. Detailed procedural results are presented in Table 2.

### 3.1. MVARC 30-Day Results

**Figure 2:**
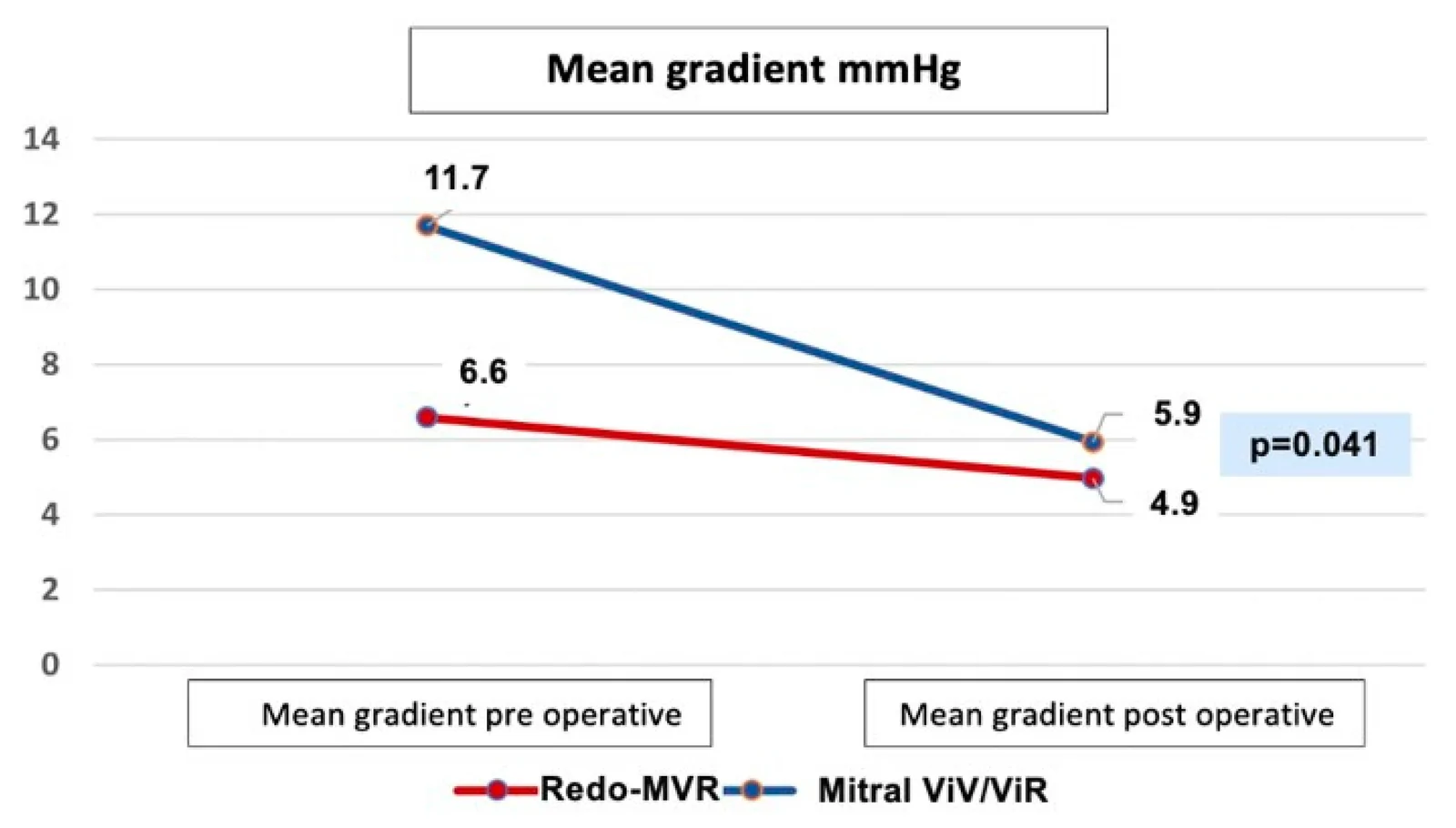
Mean transvalvular gradient before and after the procedure in the ViV/ViR and redo-MVR groups (*p* = 0.041).

At 30 days, device success (as defined in Section 2.2) was achieved in 40.0% (6/15) of patients in the ViV/ViR group and in 86.7% (13/15) of patients in the redo-MVR group (*p* = 0.02). Procedural success, which required device success plus freedom from major device- and procedure-related adverse events, was achieved in 40.0% (6/15) of ViV/ViR patients and in 66.7% (10/15) of redo-MVR patients (*p* = 0.27) and, by definition, could not exceed device success. Device success was not achieved in 9 ViV/ViR patients and 2 redo-MVR patients. The reasons (Table 3) were increased transvalvular gradients (mean gradient ≥ 5 mmHg) and access site-related vascular or structural complications, and patients could present with more than one reason. In redo-MVR, device failure denotes early failure of the newly implanted surgical prosthesis or its surgical access to meet the MVARC criteria within 30 days. One ViV/ViR patient (6.7%) experienced a TIA and one (6.7%) a stroke, while one redo-MVR patient (6.7%) suffered a stroke (*p* = 1.00). Prolonged mechanical ventilation (>72 h) occurred in one patient in each group. There were no significant differences in intensive care unit stay (*p* = 0.52) or total postoperative hospital stay (*p* = 0.23). Bleeding, according to the MVARC bleeding scale, was significantly more common and more extensive after redo-MVR (*p* = 0.03). The number of packed red blood cell units transfused within 24 h postoperatively tended to be lower after ViV/ViR (0.4 ± 0.2 vs. 1.8 ± 0.7 units, *p* = 0.07). Rates of acute renal failure stage II/III and permanent pacemaker implantation were comparable between groups (*p* = 1.0). No patient in either group had relevant mitral regurgitation (grade II/III) at discharge. Mean transvalvular gradients at discharge were higher in the ViV/ViR group (5.9 ± 1.5 mmHg vs. 4.9 ± 0.8 mmHg, *p* = 0.04). Mean transvalvular gradient before and after the procedure in the ViV/ViR and redo-MVR groups is pictured in Figure 2 (*p* = 0.041).

### 3.2. MVARC Results During Follow-Up

Survival follow-up at 365 days was complete for all patients, while clinical and echocardiographic follow-up data were available for 53.3% of ViV/ViR patients and 66.7% of redo-MVR patients. Patient success at one year was observed in 75% of ViV/ViR patients and 60% of redo-MVR patients. Cardiac-related rehospitalization occurred in one patient in each group after 6 months. No valve migration or valve thrombosis was observed. One ViV/ViR patient developed endocarditis 1034 days after the procedure, and one redo-MVR patient developed endocarditis 623 days after surgery. Clinical and echocardiographic follow-up was incomplete. One-year patient success was only fully assessed in 8/15 ViV/ViR patients and 10/15 redo-MVR patients. Endpoint-specific denominators are therefore reported explicitly in Table 4.

### 3.3. Descriptive Outcomes of ViV and ViR

This section describes the 15 patients of the transcatheter cohort separately according to the type of failed substrate (13 valve-in-valve [ViV] and 2 valve-in-ring [ViR] procedures. (Table 5).

**Figure 3:**
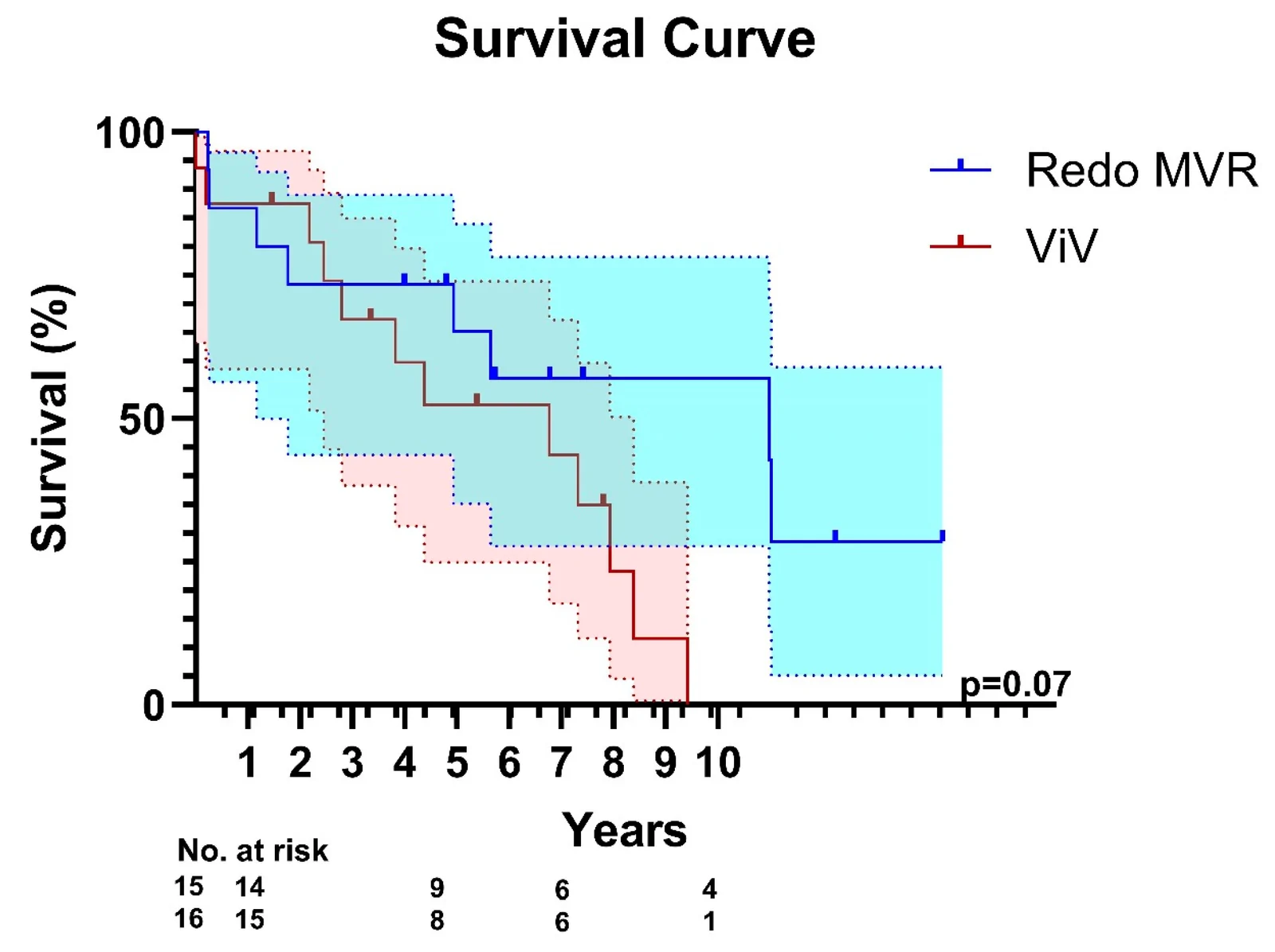
Kaplan–Meier survival curves during follow-up for the ViV/ViR and redo-MVR groups.

Kaplan–Meier analysis (Figure 3) demonstrated comparable early and mid-term survival between the ViV/ViR and redo-MVR groups, with overlapping survival curves up to approximately 6 years of follow-up. Beyond this time point, survival rates began to diverge, with the ViV/ViR group showing a more pronounced decline compared to redo-MVR. At longer-term follow-up, the redo-MVR group maintained a relatively stable survival probability, whereas survival in the ViV/ViR group decreased steadily after 6–7 years. However, this difference did not reach statistical significance in the log-rank test (*p* = 0.07).

## 4. Discussion

The operative risk of repeated open procedures on other heart valves is considered to be lower compared to redo-MVR for bioprosthesis failure [13]. Modern practice favors a valve replacement technique with preservation of the native holding apparatus when reconstruction is not an option in order to best preserve cardiac geometry and prevent ventricular dilatation. Patients who are elderly, have kidney disease, or have reduced left ventricular function are at particular risk for morbidity and mortality and may benefit from alternate, transcatheter-based therapy choices [14]. Mitral ViV/ViR interventions are increasingly performed as part of routine clinical care, but few directly comparable data presenting long-term outcomes in current cohorts have been reported.

In this single-center experience, we present favorable results for both techniques, catheter-based and surgical treatment. The overall mortality was lower than expected in both cohorts, with an expected mortality of 12% according to EuroSCORE II and an observed mortality of 6.7% in ViV/ViR, and an expected mortality of 10% and an observed mortality of 0% in redo-MVR. The main finding of our study was that device success differed significantly between the two cohorts. However, this finding should be interpreted with caution, as the cohorts differed substantially in their baseline characteristics, including age, renal function, and the type of previous mitral valve intervention (bioprosthesis versus annuloplasty ring). A previously reported limitation of ViV/ViR is the occurrence of elevated postoperative transvalvular gradients.

### 4.1. Baseline Characteristics

Although there was no significant difference between ViV/ViR and redo-MVR patients in terms of their risk scores, ViV/ViR patients’ risk profiles tended to be higher, particularly because renal function was more often impaired and patients were older. ViV/ViR treatments have given patients with deteriorated bioprostheses or failed repair an alternative therapeutic choice, which may have resulted in the increased referral of higher-risk patients. This seems plausible when considering that as many ViV/ViR patients could be enrolled in a much shorter period of time than redo-MVR patients over 20 years of surgery. A so-called “On Top Recruitment Phenomenon” has already been described for other catheter-based interventions on the mitral and aortic valve [15].

### 4.2. Indications for Redo-MVR and ViV/ViR

The interdisciplinary heart team should evaluate each patient prior to surgery. The decision to perform a surgical operation or another type of intervention should therefore be carefully considered in light of preoperative diagnostics and risk factors. Patients at high risk of left ventricular outflow tract obstruction (LVOTO), endocarditis, or thrombosis of the prosthetic valves, as well as those with paravalvular leakage that cannot be closed percutaneously, should receive redo-MVR. In cases of small or rigid rings and small bioprostheses, redo-MVR should also not be omitted as an option. This remains true for patients who require additional combined cardiac surgery (e.g., aortocoronary bypass grafting, tricuspid valve procedures, aortic procedures, etc.). There is one strong anatomical reason against ViV/ViR: the potential risk of LVOTO. This risk can be evaluated by differentiated preoperative CT planning, and, if necessary, the interdisciplinary heart team can decide against ViV/ViR if the risk is found to be greatly increased [16].

The aorto-mitral annulus angle (AMA) is an important predictor of post-procedural LVOTO. No obstruction was observed at 180°, whereas complete obstruction occurred at 90° [17]. Notably, LVOT area reduction increased exponentially between AMA angles of 135° and 120°. In our cohort, a proportion of patients was treated via the transapical access route. While this was the case for historical reasons, transseptal ViV/ViR should be used whenever possible, since transapical ViV/ViR is associated with increased mortality and morbidity compared with transseptal ViV/ViR [18].

### 4.3. Procedural Results and MVARC 30-Day Results

Mitral valve re-operations are generally expected to have a 30-day mortality of 5% to 15%, with adverse events such as major bleeding and atrial arrhythmias being much more common. While transapical approaches are still used, the less invasive transseptal technique is increasingly applied for mitral ViV/ViR procedures. According to the VIVID registry (2023), the transseptal approach is now used in approximately 74.6% of mitral ViV procedures, reflecting the shift toward a fully percutaneous and less invasive technique. Procedural success remains high (~96.4%), though residual mitral stenosis with mean gradients ≥ 5 mmHg was observed in roughly half of patients. Severe complications such as LVOTO were rare (~3.2%), but procedural outcomes were influenced by device positioning, with asymmetric implantations more frequently associated with residual gradients. This underscores that, alongside the access route, implantation technique and valve positioning are key determinants of procedural risk and hemodynamic performance. At our institution, mitral ViV/ViR procedures are still performed transseptally when indicated, which may help reduce procedural risk, though surgical revision remains our preferred approach whenever feasible [19,20].

Although technical success was high and comparable, device success was significantly higher after redo-MVR, mainly due to elevated postoperative gradients in the ViV/ViR group. Increased transmitral gradients were the most relevant ViV/ViR-specific complication, leading to device failure in seven cases, despite a reduction from 11.7 to 5.9 mmHg. These gradients are likely related to smaller implanted prostheses within prior valves or rings. In patients with small prostheses, reoperation should be considered to avoid mismatch, although elevated gradients in the mitral position appear less critical than in the aortic position [21,22]. A mean gradient of ≥5 mmHg, which was frequently observed in our study, is considered “device failure” according to MVARC criteria. We found a significant difference in mean gradients after ViV/ViR and redo-MVR, consistent with previous studies on ViV interventions for bioprostheses in the mitral valve position. In our experience, the mean gradients after ViV/ViR were lower than in previous publications (5.9 vs. 6.9 mmHg), probably because larger prostheses were implanted in our cohort (28 mm vs. 27.1 mm in the Valve-in-Valve International Data [VIVID] registry) [23]. Although the therapeutic significance of higher gradients following ViV/ViR, particularly in elderly patients, is unknown, a longer-term study may be necessary given the possibility that prosthetic valve durability may be compromised.

Moderate-to-severe regurgitation was observed in 16.6% (ViR) and 12% (ViV) of patients in the VIVID registry, but grade II/III regurgitation was not observed in our study, either in ViV or ViR patients [22]. However, in the cohort presented here, the proportion of ViR patients was lower than in the VIVID cohort, at 13.3% (2/15). ViR is known to have a higher incidence of residual paravalvular insufficiency due to the ovality of annuloplasty rings compared with ViV inside circular bioprostheses. There are also differences between ring types, as they may be flexible, rigid, or semi-rigid. Given the high-risk profile of our ViV/ViR patients, with a mean logistic EuroSCORE II of 12.0% and the likelihood of a learning curve, the 30-day mortality rate of 6.7% seems reasonable given that this study included early cases and that in 40% of cases, as discussed above, access was via a transapical route.

The fatality observed in this cohort was caused by a post-procedural pericardial effusion that was hemodynamically significant and required emergency revision surgery, as the bleeding did not cease. Transseptal access was initially used to perform ViV/ViR in this patient.

The rate of LVOTO in mitral ViV is reported to be 2.2% and in ViR 5.5% (VIVID data). No patient in our ViV/ViR cohort experienced LVOTO.

In redo-MVR, most complications were related to the access site and bleeding. Nevertheless, as no patient in our redo-MVR group died within 30 days, we perceived the mortality to be lower than in previously described redo-MVR publications [13,14].

In a retrospective multicenter study including 10 cardiac centers in Germany from 2014 to 2019, 30-day and mid-term mortality were assessed in 273 patients (193 of whom underwent redo-MVR), demonstrating that observed mortality was lower than expected in both groups [24]. Notably, outcomes after redo-MVR are likely influenced by institutional and operator experience, with lower mortality reported in high-volume centers and when procedures are performed by experienced surgeons. In addition, minimally invasive cardiac surgery approaches may represent a valuable option in the reoperative setting, potentially reducing surgical trauma and improving perioperative outcomes.

A thorough understanding of both therapeutic choices, including their benefits, drawbacks, and associated risks, greatly enhances the heart team’s ability to make decisions and determine the best course of treatment for each patient. For this reason, international guideline recommendations have incorporated the heart team’s decision for such patients [25]. Overall, the current work demonstrates that both therapy techniques can produce favorable results with proper preoperative planning and indication.

### 4.4. MVARC Results During Follow-Up

Kaplan–Meier survival analysis demonstrated a trend toward improved long-term survival in the redo-MVR group compared with the ViV/ViR group, but patients in the redo-MVR group were younger and therefore had a higher baseline probability of survival. Early and mid-term survival up to 6 years was comparable between both groups; however, a progressive separation of the curves was observed during follow-up. From approximately 6–7 years onward, the ViV/ViR group showed a more pronounced decline in survival, which is plausible given that these patients were on average 10 years older (log-rank test: *p* = 0.07). Kaplan–Meier survival analysis demonstrated a descriptive trend toward lower long-term survival in the ViV/ViR group after approximately 6–7 years. This finding must be interpreted in the context of the substantially older age of the ViV/ViR cohort and should not be interpreted as evidence of a treatment effect.

### 4.5. Limitations

This study has several limitations. It is retrospective in nature, which carries inherent disadvantages. Due to the limited number of accessible patients in this relatively newly growing population, one-to-one matching was not possible. As a result, the comparison groups are heterogeneous, making it difficult to fully compare the outcomes of the two methods. The small sample size further complicates statistical analysis and limits its generalizability. Because of the small single-center cohort, neither subgroup analyses nor risk stratification were feasible, and larger multicenter datasets would be required for this purpose. In addition, the MVARC endpoints were developed for transcatheter mitral interventions; their uniform application to the surgical cohort allows a consistent comparison but has not been formally validated for redo surgery. The present study cannot establish which therapeutic approach is preferable to the other, but it can help the interdisciplinary heart team in patient selection. Due to the limited follow-up time, long-term results could not be fully compared. This study has several limitations. In addition to its retrospective nature and small sample size, the comparison is subject to substantial selection bias because treatment allocation was not randomized and the cohorts differed markedly in age, renal function, and underlying failed mitral valve substrate. ViV/ViR patients were predominantly patients with failed bioprostheses, whereas redo-MVR patients more frequently had failed annuloplasty rings. Incomplete clinical and echocardiographic follow-up further limits interpretation of the one-year endpoints. Accordingly, the present study should be regarded as descriptive and hypothesis-generating rather than as evidence of comparative treatment effectiveness.

## 5. Conclusions

Despite older age and more comorbidities, ViV/ViR showed comparable 30-day mortality and similar neurological event rates to redo-MVR. Higher postoperative gradients were the main limitation of ViV/ViR, while bleeding complications were more frequent after redo-MVR. One-year patient, procedural, and technical success were comparable. Long-term survival showed a non-significant trend favoring redo-MVR. Overall, ViV/ViR is an attractive alternative, and both approaches should be considered complementary, with treatment decisions guided by a multidisciplinary heart team. In this small retrospective single-center cohort, ViV/ViR and redo-MVR showed different procedural profiles. ViV/ViR was associated with higher postoperative transmitral gradients, whereas bleeding complications were more frequent after redo-MVR. Because of substantial baseline differences, including age, renal function, and the underlying failed valve/ring substrate, these findings should be interpreted as descriptive and hypothesis-generating rather than as evidence of comparable or equivalent treatment effectiveness. Both approaches may represent complementary options in appropriately selected patients, with treatment decisions guided by a multidisciplinary heart team.

## Figures and Tables

**Table 1:** Baseline characteristics.

Baseline Characteristics	ViV/ViR (*n* = 15)	Redo-MVR (*n* = 15)	*p*-Value
Age, years ± SD	76.9 ± 5.8	68.0 ± 8.4	<0.001
Female gender	7 (46.7%)	10 (66.7%)	0.462
Logistic EuroSCORE II, % ± SD	12.0 ± 2.9	10.0 ± 2.7	0.060
NYHA class III/IV, n	15 (100%)	15 (100%)	1.000
Peripheral vascular disease	4 (26.7%)	3 (20%)	1.000
GFR, mL/min/1.73 m^2^ ± SD	42.1 ± 13.5	60.8 ± 11.6	<0.010
Previous stroke/TIA	3 (20.0%)	1 (6.7%)	0.348
EF 30–50%	2 (13.3%)	4 (26.7%)	0.919
EF < 30%	3 (20%)	2 (13.3%)	
Degenerated bioprosthesis	13 (86.6%)	3 (20.0%)	<0.010
Failed annuloplasty ring (St. Jude and Carpentier ring)	2 (13.3%)	12 (80.0%)	
Relevant coronary artery disease	9 (60.0%)	9 (60.0%)	1.000
Previous CABG	4 (26.7%)	6 (40.0%)	0.473
Time since MV surgery, years ± SD	9.9 ± 4.4	4.2 ± 5.1	<0.010
Type of degeneration: stenosis	2 (13.3%)	1 (6.7%)	0.182
Regurgitation	6 (40%)	11 (73.3%)	
Mixed	7 (46.7%)	3 (20%)	
Mean gradient MV, mmHg ± SD	11.7 ± 8.8	6.6 ± 4.1	0.050

GFR = glomerular filtration rate; TIA = transient ischemic attack; CABG = coronary artery bypass grafting; MV = mitral valve; SD = standard deviation; NYHA = New York Heart Association; EF = ejection fraction. Redo-MVR = redo surgical mitral valve replacement (including patients with a previous annuloplasty ring); ViV/ViR = valve-in-valve/valve-in-ring.

**Table 2:** Procedural results.

Result of the Procedure	ViV/ViR (*n* = 15)	Redo-MVR (*n* = 15)	*p*-Value
Access: Transapical	6 (40%)	n/a	n/a
Transfemoral	9 (60%)	n/a	
(Re-)sternotomy	n/a	14 (93.3%)	
Anterolateral minithoracotomy	n/a	1 (6.7%)	
Transcatheter heart valve inserted: Sapien 3	12 (80%)	—	n/a
Sapien XT	3 (20.0%)	—	
Implanted valve size, mm ± SD	28.2 ± 1.4	29.5 ± 1.8	0.029
Cardiopulmonary bypass time, min ± SD	n/a	159.4 ± 57.1	n/a
Aortic cross-clamp time, min ± SD	n/a	99.7 ± 35.0	n/a
Postoperative need for mechanical circulatory support: IABP	0	1 (6.7%)	1.000
ECMO	1 (6.7%)	2 (13.3%)	1.000

n/a = not available; IABP = intra-aortic balloon pump; ECMO = extracorporeal membrane oxygenation; SD = standard deviation; mm = millimeters; min = minutes.

**Table 3:** Thirty-day results (MVARC).

30-Day Results (MVARC)	ViV/ViR (*n* = 15)	Redo-MVR (*n* = 15)	*p*-Value
30-day mortality	1 (6.7%)	0	1.00
Procedural success after 30 days (device success + absence of major procedure- and prosthesis-related adverse events)	6/15 (40%)	10/15 (66.7%)	0.27
**Device success after 30 days**	**6 (40.0%)**	**13 (86.7%)**	**0.02**
Reason for not achieving device success			
Increased gradient	7	3	
Migration of the valve	0	0	
Paravalvular leakage (>1)	0	0	
Access site-related	4	2	
Surgery/death/stroke/LVOT obstruction	0	0	
Neurological events			1.00
TIA	1 (6.7%)	0	
Stroke	1 (6.7%)	1 (6.7%)	
Prolonged mechanical ventilation (>72 h)	1 (6.7%)	1 (6.7%)	1.00
Length of ICU stay, nights, median (IQR)	3.0 (1.0–5.0)	2.0 (1.75–16.0)	0.52
Length of postoperative hospital stay, days, median (IQR)	9.0 (5.0–18.0)	16.5 (13.3–44.8)	0.23
Access site-related cardiac structural complications	5 (33.3%)	6 (40.0%)	1.00
Re-exploration for bleeding	4 (26.7%)	5 (33.3%)	1.00
**MVARC bleeding scale:** **none**	**7 (46.6%)**	**1 (6.7%)**	**0.03**
Minor	3 (20%)	9 (60%)	
Major	1 (6.7%)	1 (6.7%)	
Extensive	3 (20%)	1 (6.7%)	
Life-threatening	1 (6.7%)	3 (20%)	
Fatal	0	0	
Transfused PRBC units/24 h postoperative, mean ± SEM	0.4 ± 0.2	1.8 ± 0.7	0.07
Acute renal failure stage II/III	1 (6.7%)	2 (13.3%)	1.00
Permanent pacemaker implantation	3 (20.0%)	4 (26.7%)	1.00
Mitral regurgitation grade II/III at discharge	0	0	1.00
Mean gradient at discharge, mmHg ± SD	5.9 ± 1.5	4.9 ± 0.8	0.04

IQR = interquartile range; SD = standard deviation; SEM = standard error of the mean; mmHg = millimeters of mercury; PRBCs = packed red blood cells; TIA = transient ischemic attack.

**Table 4:** Results during follow-up.

Results During Follow-Up (MVARC) *	ViV/ViR	Redo-MVR	*p*-Value
Cardiac-related rehospitalization after 6 months	1/15 (6.7%)	1/15 (6.7%)	1.00
NYHA class I/II after 6 months	13/15 (86.6%)	14/15 (93.3%)	1.00
Additional stroke/TIA	1/15 (6.7%)	0/15	1.00
Valve thrombosis	0/8	0/10	1.00
Valve migration	0/8	0/10	1.00
Endocarditis	1/15 (6.7%)	1/15 (6.7%)	
Patient success at 1 year	6/8 (75%)	6/10 (60%)	0.64
Time to endocarditis, days	1034	623	

* Results are indicated only for patients for whom one-year follow-up is available. Due to incompleteness of data, not all MVARC one-year endpoints could be reported. NYHA = New York Heart Association; TIA = transient ischemic attack.

**Table 5:** Descriptive outcomes of ViV and ViR.

Outcomes (MVARC)	ViV (*n* = 13)	ViR (*n* = 2)
Technical success	10/13 (76.9%)	1/2 (50.0%)
Device success at 30 days	6/13 (46.2%)	0/2 (0%)
Procedural success at 30 days	6/13 (46.2%)	0/2 (0%)
30-day mortality	0/13 (0%)	1/2 (50.0%)
Neurological events	2/13 (15.4%)	0/2 (0%)
TIA	1/13 (7.7%)	0/2 (0%)
Stroke	1/13 (7.7%)	0/2 (0%)
Prolonged mechanical ventilation > 72 h	1/13 (7.7%)	0/2 (0%)
Access site-related cardiac structural complications	4/13 (30.8%)	1/2 (50.0%)
Re-exploration for bleeding	3/13 (23.1%)	1/2 (50.0%)
Acute renal failure stage II/III	4/13 (30.8%)	0/2 (0%)
Permanent pacemaker	4/13 (30.8%)	0/2 (0%)
MR grade II/III at discharge	0/13 (0%)	0/2 (0%)
Mean gradient at discharge, mmHg ± SD	6.1 ± 1.5	6.4 ± 2.1

ViV = valve-in-valve; ViR = valve-in-ring; TIA = transient ischemic attack; MR = mitral regurgitation; MVARC = Mitral Valve Academic Research Consortium; SD = standard deviation.

## Data Availability

The data presented in this study are available upon request from the corresponding author due to privacy and ethical restrictions relating to the patient cohort.

## Abbreviations

The following abbreviations are used in this manuscript:

AMA	Aorto-mitral annulus angle
BMI	Body mass index
CABG	Coronary artery bypass grafting
COPD	Chronic obstructive pulmonary disease
EF	Ejection fraction
ICU	Intensive care unit
Logistic EuroSCORE	Logistic European System for Cardiac Operative Risk Evaluation
LVOT	Left ventricular outflow tract
LVOTO	Left ventricular outflow tract obstruction
MV	Mitral valve
MVARC	Mitral Valve Academic Research Consortium
NYHA	New York Heart Association
Redo-AVR	Redo aortic valve replacement
Redo-MVR	Redo mitral valve replacement (surgical explantation of a failed bioprosthesis or annuloplasty ring and implantation of a new prosthesis)
THV	Transcatheter heart valve
TIA	Transient ischemic attack
ViV	Valve-in-valve
ViR	Valve-in-ring
TAVI	Transcatheter aortic valve implantation
VIVID	Valve-in-Valve International Data
